# Exposure to diesel particulates induces an immunosuppressive microenvironment that promotes the progression of lung cancer

**DOI:** 10.1016/j.neo.2025.101255

**Published:** 2025-11-21

**Authors:** Marie-Laure Delhez, Maëlle Bosmans, Lucia Rodriguez Rodriguez, Alison Gillard, Silvia Blacher, Arnaud Blomme, Pierre Close, Bénédicte Machiels, Marie-Julie Nokin, Didier Cataldo

**Affiliations:** a Laboratory of Tumour and Development Biology, GIGA Institute, University of Liège, Hippocrates Avenue, 1, B23, 4000 Liège, Belgium; b Department of Infectious and Parasitic Diseases, Faculty of Veterinary Medicine - FARAH, University of Liège, Quartier Vallée 2, Avenue de Cureghem 6, B43a, 4000 Liège, Belgium; c Laboratory of Cancer Signaling, GIGA-Institute, University of Liège, Hippocrates Avenue, 1, B34, 4000 Liège, Belgium; d WELBIO Department, WEL Research institute, Avenue Pasteur, 6, 1300 Wavre, Belgium; e Department of Respiratory Diseases, CHU Liège and University of Liège, Hippocrates Avenue, 1, B23, 4000 Liège, Belgium

**Keywords:** Air pollution, Lung cancer, Neutrophils, NETs, PMN-MDSC, Immunity

## Abstract

A comprehensive understanding of the mechanisms by which air pollutant exposure drives cancer progression remains incomplete. Particulate matter has been shown to induce genotoxicity and mutagenesis through oxidative stress both *in vivo* and *in vitro*. However, its impact on the pulmonary immune microenvironment and its role in modulating anti-tumour immune responses remains poorly characterized.

Here, we report that chronic exposure to diesel exhaust particles (DEPs), a major component of PM2.5, induces an immunosuppressive lung microenvironment that promotes tumour progression in a KRAS-driven lung adenocarcinoma model (*Kras^LSL-G12D/+^-Trp53^lox/lox^* or KP mice). This environment is characterized by the emergence of PMN-MDSC (CD14^pos^ PMNs) that exhibit NET formation and an immunosuppressive gene expression and functional profile. Additionally, we observed increased infiltration of regulatory T cells (Tregs), and upregulation of exhaustion/activation and immunosuppressive markers on T cells, factors that likely contribute to the increased tumour burden and enhanced tumour cell proliferation seen in DEP-exposed KP mice.

Our study reveals how chronic DEP exposure reshapes the lung microenvironment in ways that may impair the ability to mount effective anti-tumour immune responses. These findings highlight the need for stronger public and occupational health policies aimed at reducing air pollution and its associated disease burden.

## Highlights

• Kras mutated mice exposed to DEP develop larger, more proliferative lung tumours.
• Exposure to DEP recruits CD14^pos^ PMNs in lungs.
• CD14^pos^ PMNs display suppressive features and induce T cell dysfunction.
• DEP-induced recruitment of immunoregulatory PMNs might be relevant in humans.

## Introduction

Air pollution is a major environmental and public health concern. Particulate matter (PM), classified by aerodynamic size, is a major airborne pollutant that penetrates deep into the lungs and is linked to multiple adverse effects, such as cardiovascular diseases, strokes and lung cancer [1]. Epidemiological studies conducted on large cohorts have established a correlation between exposure to inhaled fine particles measuring ≤2.5 µm (PM2.5) and all-cause mortality [2]. Diesel exhaust particles (DEP) are a key component of PM2.5 in air pollution, and they have been classified as class 1 carcinogens to humans by the International Agency for Research on Cancer (IARC) [3]. However, beyond these well-established mechanisms, the influence of DEP on the pulmonary immune microenvironment, and its potential interference with anti-tumour immune responses remains poorly understood.

Polymorphonuclear neutrophils (PMNs) identified in mice as Ly6G+Ly6C+CD11b+ cells represent the most abundant population of innate immune cells in circulation. They play a central role in the early response to pathogens through phagocytosis, degranulation and NET formation. In addition to their antimicrobial functions, PMNs also contribute to immune regulation by producing cytokines and chemokines and engaging in crosstalk with other immune cell populations [4]. DEP exposure induces the recruitment of PMNs to the lung, where they play a key role in the pathogenesis of air pollution-related diseases, notably by promoting the formation of neutrophil extracellular traps (NETs) [5]. Whereas PMNs are primarily involved in host defence against pathogens and tissue remodelling, their regulatory role on adaptive immunity has received growing attention in recent years. PMNs play a dual role in cancer: while some subsets contribute to anti-tumour immunity, others can promote tumour progression and suppress T cell responses [6]. The immunosuppressive activity of PMNs has been largely attributed to polymorphonuclear myeloid-derived suppressor cells (PMN-MDSCs), a subset of pathologically activated PMNs that accumulate in cancer. These cells have been shown to dampen immune responses and contribute to the formation of an immunosuppressive microenvironment, which may facilitate immune evasion and tumour progression [7]. Transcriptomic analyses have identified CD14, recognized as a marker of monocytic cells, as a signature associated with the immunosuppressive potential of PMNs in mice, distinguishing specific neutrophil subsets. The expansion of these CD14^pos^ PMN-MDSCs plays a key role in immunosuppression by limiting T cell proliferation [8]. This inhibition drives T lymphocytes into a dysfunctional state in which they lose their ability to mount effective immune responses. We hypothesized that DEP exposure may contribute to an immunosuppressive environment that promotes lung tumour progression. To address this hypothesis, we employed acute DEP exposure in wild-type (wt) mice and chronic DEP exposure in *Kras^LSL-G12D/+^-Trp53^lox/lox^* mice (KP mice), in which we also evaluated changes in spontaneous tumour development. We characterized the heterogeneity of PMNs recruited in the lung after acute and chronic DEP exposure and assessed their immunosuppressive phenotype using flow cytometry and functional assays. In parallel, we analysed the phenotype of infiltrating T cells to evaluate the broader impact of these changes on the tumour immune microenvironment. Given the rising global burden of air pollution and lung cancer, elucidating how DEP exposure alters the immune landscape is crucial for uncovering mechanisms that drive tumour progression and for identifying therapeutic strategies. These may include limiting the recruitment of immunosuppressive neutrophils, adapting treatments to PM-induced immune alterations, or protecting patients from further pollutant exposure during therapy.

## Materials and methods

### Mice

Animal procedures were conducted in accordance with the Federation of European Laboratory Animal Science Associations (FELASA) and all experiments had previously been approved by the Animal Ethical Committee of the University of Liege (protocol references: # 2136). C57BL/6 mice (male and female, 6–8-week-old) were purchased from Janvier’s Labs (France). *Kras^LSL-G12D/+^-Trp53^lox/lox^* (KP) mice were obtained from Dr Pierre Close’s laboratory at University of Liège (male and female mice aged 12-16 weeks were used). All mice were housed and bred in institutional specific pathogen-free facilities at the GIGA Institute (Liège, Belgium), maintained in a 12-hour light-dark cycle and had access to normal chow diet and water ad libitum.

### Intratracheal instillation of DEP into mouse lungs

Details are provided in the online supplementary material and methods section.

### Lung cell and spleen cell isolation

Details are provided in the online supplementary material and methods section.

### T cell isolation from lymphoid tissues

Lymphocytes were isolated by mechanically disrupting lymph nodes from naïve C57BL/6 mice using a flat-bottomed syringe, followed by collection in PBS-EDTA. Pan T cells were then isolated using the Pan T Cell Isolation Kit (Miltenyi Biotec) in accordance with the manufacturer's instructions, and separation was performed using LS columns on a QuadroMACS separator (Miltenyi Biotec).

### Flow cytometry staining

Cells were first incubated for 15 min with Fc Block (anti-mouse CD16/32, clone 93, BioLegend) diluted in FACS buffer (PBS with 0,5 % BSA). Surface marker antibodies were resuspended in FACS buffer (PBS with 0,5 % BSA) and cells were incubated for 30 min with the corresponding fluorochrome-conjugated antibodies (listed in the key resources table). Cells were washed with PBS and stained with fix viability staining Zombie Aqua, Violet, or NIR (BioLegend) resuspended in PBS and incubated for 20 min. Cells were then washed with PBS and resuspended on FACS buffer or follow intracellular staining of target proteins.

For intracellular staining of cytoplasmic proteins, cells were fixed and permeabilized using Cyto-Fast Fix/Perm buffer (BioLegend) according to the manufacturer’s instructions. Antibodies were diluted in perm buffer and incubated with the cells for 30 minutes.

For intranuclear staining of proteins, cells were fixed and permeabilized using Foxp3/transcription factor staining kit (eBioscience) according to the manufacturer’s instructions. Antibodies were diluted in the permeabilization buffer of the kit and incubated with the cells for an hour.

Flow cytometry acquisition was performed on BD LSR Fortessa using FACSDiva software, and the data was processed with Flow Jo software. Gating strategies to identify the different cell populations are provided in Figure S1-S3.

### Isolation of CD14^neg^ and CD14^pos^ PMNs

Mouse neutrophils (PMNs) were isolated from lung single-cell suspension prepared as previously described. The cell suspension was incubated with Anti-Ly-6 G Microbeads (Miltenyi Biotec) and passed through MACS LS columns (Miltenyi Biotec) on a QuadroMACS separator (Miltenyi Biotec) to capture neutrophils, following the manufacturer's protocol. Neutrophils were eluted by gently plunging the LS columns and subsequently stained with anti-Ly6G, anti-CD14 and the viability marker SYTOX^TM^ blue. They were then sorted using a BD FACS Aria (BD Biosciences) as CD14 negative or positive neutrophils. The isolated PMNs were then used for further analysis.

### Cytospin of neutrophils

After FACS sorting of CD14^neg^ and CD14^pos^ PMNs, cytospins were prepared using a StatSpin CytoFuge 2 (IRIS compagny). In detail, 2.5 × 10^4^ cells were centrifuged (4400 rpm, 2 min) onto Superfrost microscope slides (Epredia) and dried overnight at room temperature. Cells were then fixed in methanol and stained with hematoxylin and eosin (H&E). Image documentation was performed using the NanoZoomer 2.0-HT slide scanner system (Hamamatsu) and the NanoZoomer Digital Pathology software.

### T cell suppression assay

CD14^neg^ and CD14^pos^ PMNs were FACS sorted as described above. T cells were isolated as previously described, then washed in PBS and labelled with CellTrace Violet (Invitrogen) following the manufacturer’s instructions. To activate the T cells in culture, a 96-well tissue culture plate was pre-coated with anti-mouse CD3 antibody (5 µg/ml; eBioscience) overnight at 4°C, then washed with PBS. Neutrophils and T cells were co-cultured at a 1:1 ratio or alone as controls in 200 µl RPMI medium (Lonza) per well, supplemented with 10 % FBS (Gibco), 50 U/ml penicillin-streptomycin (Gibco), 1 % MEM non-essential amino acids (Gibco), 1 mM sodium pyruvate (Gibco), and 0.05 mM 2-mercaptoethanol (Gibco). Anti-mouse CD28 (1 µg/ml; eBioscience) was also added to the culture to provide co-stimulatory signalling on T cells. The cells were incubated at 37°C in a 5 % CO2 environment for 72 hours, after which T cells were collected from the supernatant, stained for surface markers (anti CD3 and CD8) and viability. Finally, proliferation of CD8^+^ *T* cells was assessed using a BD LSR Fortessa flow cytometer.

### Quantitative real time polymerase chain reaction (qRT-PCR)

Details are provided in the online supplementary material and methods section.

### Identification of NETs In Vivo and In Vitro

Details are provided in the online supplementary material and methods section.

### Collection of bronchoalveolar lavage fluid (BALF)

After sacrifice, a bronchoalveolar lavage was performed by injecting 3 × 1 ml of PBS-EDTA 0,05 mM into lungs. Collected bronchoalveolar lavage fluid (BALF) was centrifuged 10 min at 110 g and supernatants were stored at −80°C for further analyses.

### Immunohistochemistry

Details are provided in the online supplementary material and methods section.

### Statistical analysis

All statistical analyses were performed using GraphPad Prism software. Results were expressed as means ± SEM. An unpaired t-test was used to compare two groups with normally distributed data. When the assumption of normality was not met, the non-parametric Mann–Whitney test was used instead. For comparisons of means between more than two experimental groups, one-way analysis of variance (ANOVA) was used as appropriate. P value > 0,05 was considered not significant (ns); P values are provided within each figure legend, together with the statistical test performed for each experiment.

## Results

**Chronic exposure to DEP favours tumour progression in a genetically engineered mouse model of lung adenocarcinoma**

**Fig. 1:**
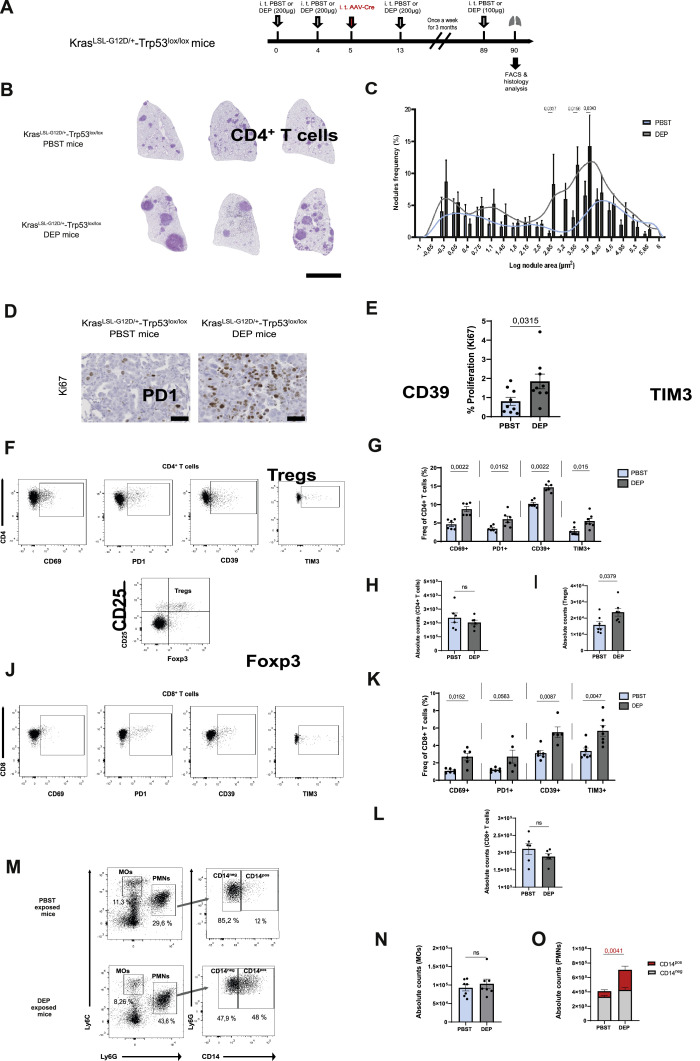
**Chronic exposure to DEP favours tumour progression in a genetically engineered mouse model of lung adenocarcinoma** (A) Experimental exposure protocol. (B) Representative images of HE-stained lungs and (C) nodules sizes distribution frequency in PBST and DEP treated Kras^LSL-G12D/+^-Trp53^lox/lox^ (KP) mice. Scale bar = 5 mm. Statistical analysis was performed using two-way ANOVA, followed by Sidak’s multiple comparisons test to assess pairwise differences between groups. (D) Representative images of Ki67 immunohistochemistry in lung sections from PBST and DEP-exposed KP mice. Scale bar = 50 µm (E) Quantification of cellular proliferation (Ki67) within nodules (*n* = 3 to 6 mice per group, two pooled experiments). Representative flow cytometry plots of (F) CD4+ *T* cells and (J) CD8+ *T* cells, frequency (%) of indicated markers in (G) CD4+ *T* cells and (K) CD8+ *T* cells, absolute counts of (H) CD4+ *T* cells, (L) CD8+ *T* cells and (I) Tregs present from PBST or DEP-exposed KP mice. (M) Gating strategy used for the identification of MOs, PMNs and their subsets. Absolute counts of (N) MOs and (O) CD14^neg^ and CD14^pos^ PMNs recruited in the lungs of PBST and DEP-exposed KP mice (*n* = 6-7 mice per group, one experiment). Data are presented as mean ± SEM. Statistical significance was assessed using Mann-Whitney test.

To assess the influence of DEP exposure on tumour development, we used a genetically engineered mouse model that develops lung tumours upon KRAS oncogenic activation combined with p53 loss (KP model). Tumour burden in the lungs of KP mice was assessed 90 days after AAV-Cre administration (Fig 1A).

Strikingly, we observed larger tumour nodules in DEP-exposed mice (Fig. 1B and C). Additionally, the density of Ki67+ cells within tumour nodules was higher in DEP-exposed KP mice, indicating increased tumour cell proliferation in the DEP-altered lung microenvironment (Fig. 1D and E).

Although total CD4+ and CD8+ *T* cell numbers were unchanged in the lung following DEP chronic exposure (Fig. 1H, 1L), we observed alterations in the composition and activation state of T cell populations. Specifically, there was an increase in Treg numbers (Fig. 1I), along with an upregulation of activation and exhaustion markers such as CD69, PD-1 and TIM3, as well as CD39, which contributes along with CD73 to adenosine production in the lung microenvironment, thereby promoting immunosuppression (Fig. 1F, 1G, 1J, 1K). Of note, expression of these makers on CD8+ *T* cells was less compared to CD4+ *T* cells.

While we observed no changes in the total counts of monocytes (Ly6C^high^Ly6G^-^) upon chronic DEP exposure in our KP model (Fig. 1N), we did observe a markedly increase in CD14^pos^ PMNs, a subset typically associated with PMN-MDSCs (Fig. 1O). In contrast, no significant differences were observed in the total numbers of CD14^neg^ PMNs.

### Acute exposure to DEP induces CD14^pos^ recruitment and NET formation in the lungs

**Fig. 2:**
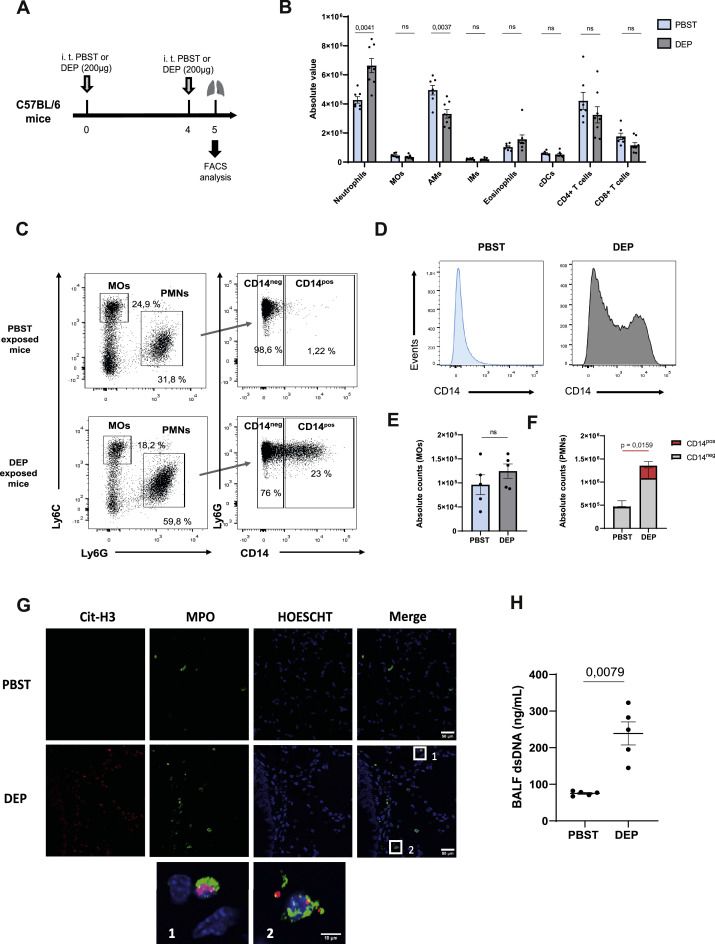
**Acute exposure to DEP induces PMNs CD14^pos^ recruitment and NET formation in the lungs** (A) Experimental exposure protocol. (B) Number of indicated immune cell populations detected by flow cytometry in the lungs after intratracheal instillations of PBST or DEP (200 µg) (*n* = 7 to 8 mice per group, one experiment). (C) Gating strategy used for the identification of MOs, PMNs and their subsets in lungs of PBST or DEP exposed mice. (D) Representative flow cytometry histogram of CD14 expression in PMNs and absolute counts of (E) MOs, (F) PMNs CD14^neg^ and CD14^pos^ recruited in the lungs of PBST or DEP exposed mice (*n* = 4 to 6 mice per group, three independent experiments were performed). (G) Representative immunofluorescence images obtained by confocal microscopy of lung sections from mice 24 h after intratracheal DEP treatment compared to PBST. Staining shows Cit-H3 (red), MPO (green) and Hoechst (blue). The higher magnification views in the inserts (1-2) show NET formation, demonstrated by the co-localization of Cit-H3 (red), MPO (green) and DNA (blue). (H) BALF levels of dsDNA were measured using a PicoGreen assay kit (*n* = 5 mice per group, one experiment). Data are presented as mean ± SEM. Statistical significance was assessed using Mann-Whitney test.

To further explore the underlying mechanisms and to dissect the specific immune alterations by which DEP exposure influence lung tumour development, we first assessed lung inflammation caused by acute DEP exposure in C57BL/6 mice (Fig. 2A) by quantifying lung immune cell populations by flow cytometry. Acute DEP exposure led to a significant infiltration of polymorphonuclear neutrophils (PMNs) and a reduction in alveolar macrophages (AMs), while monocytes (MOs), interstitial macrophages (IMs), eosinophils, conventional dendritic cells (cDCs), CD4+ and CD8+ lymphocytes remained unchanged (Fig. 2B). Interestingly, while monocyte numbers remained unaffected (Fig 2C, 2E), we observed that acute DEP exposure was sufficient to induce the emergence of a CD14^pos^ PMN subpopulation (Fig. 2C-F). Of note, the emergence of this population was restricted to the local lung environment, since no presence of CD14^pos^ PMNs was detected in the spleen despite the overall increase in PMNs following DEP exposure. (Fig. S4A, S4C).

Considering the established link between NET formation and tumour progression [4,9], we investigated whether PMNs increase NET formation after DEP acute exposure. We identified NETs in lung histological samples by the co-localization of citrullinated-histone (Cit-H3), myeloperoxidase (MPO), and DNA -key components of neutrophil granules that are released during NET formation. NET formation was observed in lung tissue of mice exposed to DEP administration but was absent in control mice (Fig. 2G). Furthermore, mice exposed to DEP showed significantly higher levels of dsDNA in their BALF as compared to the control group (Fig. 2H). These results demonstrate that acute DEP exposure triggers the recruitment of PMNs into the lung, promotes the appearance of a CD14^pos^ subpopulation, and leads to NET formation.

### DEP-recruited CD14^pos^ PMNs exhibit an immunosuppressive phenotype

**Fig. 3:**
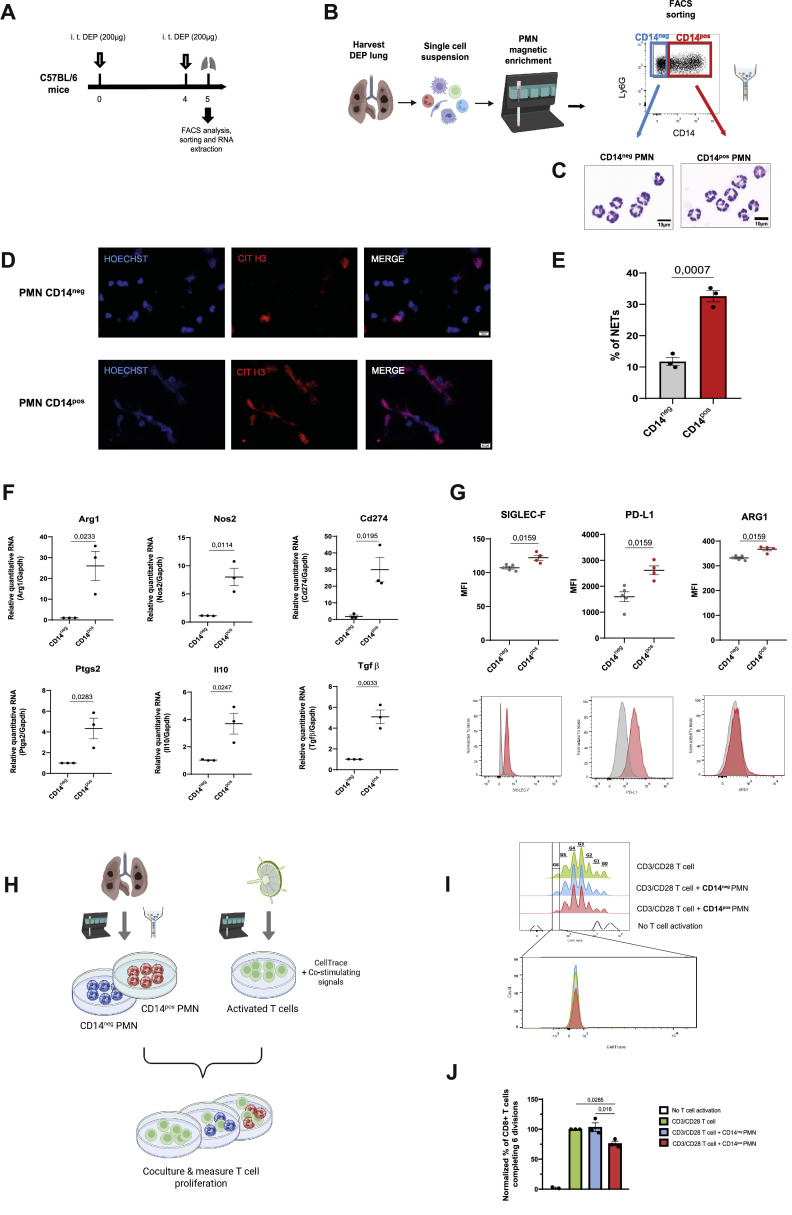
**DEP-recruited CD14^pos^ PMNs exhibit an immunosuppressive phenotype** (A) Experimental exposure protocol (B) Scheme representing of PMN isolation from DEP exposed lungs using magnetic column separation of Ly6G+ cells, followed by FACS cell sorting of CD14^neg^ and CD14^pos^ PMNs. (C) Representative cytospin images from CD14^neg^ and CD14^pos^ PMN sorted populations stained with H&E. Scale bar, 10 µm. (D) Representative images of ex vivo cultured CD14^neg^ and CD14^pos^ PMNs stained with anti-citrunillated histone 3 antibody (CIT H3; red) and Hoechst (blue). NETs are identified by the co-localization of Cit-H3 and Hoechst. Scale bar = 10 µm. (E) Percentage of NETs measured on fluorescence microscopy images of ex vivo cultured CD14^neg^ and CD14^pos^ PMNs (*n* = 5 pooled mice per experiment, one experiment) (F) Expression of indicated genes by qPCR in CD14^neg^ and CD14^pos^ PMNs from lungs of DEP exposed mice (*n* = 5 pooled mice by experiment, three independent experiments). Data were analysed using an unpaired t-test. (G) Mean fluorescence intensity (MFI) quantification and representative flow cytometry plots of selected markers in CD14^neg^ and CD14^pos^ PMNs from lungs of DEP exposed mice (*n* = 4 to 5 mice per experiment, one experiment). Statistical significance was assessed using Mann-Whitney test. (H) Scheme representing PMN-induced T cell suppression assay. T cells were isolated from lymph nodes of untreated C57BL/6 mice by negative selection using magnetic column separation, stained with Cell Trace dye and co-cultured in the presence of co-stimulatory molecules CD3/CD28 for 72 hours with CD14^neg^ and CD14^pos^ PMN isolated from DEP exposed mice lungs. (I) Representative overlay histogram showing Violet Cell Trace signals in control group with only T cells without stimulation (white), only T cells activated by anti-CD3/CD28 (green), activated T cells in presence of CD14^neg^ PMN (blue) and activated T cells in presence of CD14^pos^ PMN (red). Zoomed-in view of T cells that have undergone six divisions (below). (J) Quantitative bar chart showing the normalized percentage of CD8+ *T* cells completing 6 divisions. Each data point represents one experiment with at least 5 pooled mice per experiment. Data coming from the T cell suppression assay was analysed using a one-way ANOVA followed by Tukey’s post hoc test for multiple comparisons. Data are presented as mean ± SEM.

To elucidate the functional immunosuppressive characteristics of PMNs recruited to the lungs in acute DEP-exposed mice (Fig. 3A), we isolated PMNs based on CD14 expression (Fig. 3B). No distinct morphological differences were observed between CD14^neg^ and CD14^pos^ PMN populations (Fig. 3C). However, immunohistochemistry analysis revealed increased NET formation in CD14^pos^ PMNs compared to their CD14^neg^ counterparts (Figs. 3D, 3E). Next, we quantified in both PMN populations the expression of key genes associated with the immunosuppressive activity of MDSCs, such as Arg1, Nos2, Cd274, Ptgs2, Il-10 and Tgfβ [7]. RT-qPCR analysis demonstrated a significant upregulation of all assessed genes in CD14^pos^ PMNs compared to CD14^neg^ PMNs in DEP-exposed mice (Fig. 3F). In addition, CD14^pos^ PMNs also exhibited elevated protein expression of Siglec-F, PD-L1 and Arg1 compared to CD14^neg^ PMNs (Fig. 3G).

To functionally confirm the suppressive activity of DEP-recruited CD14^pos^ PMNs, we conducted a CD8^+^ *T* cell suppression assay. Both CD14^neg^ and CD14^pos^ PMNs isolated from DEP exposed lungs were cultured in presence of activated T cells isolated from lymph nodes of naïve mice (Fig. 3H). The CD14^neg^ PMNs exhibited no detectable suppressive activity when co-cultured with activated T cells, whereas co-culture with CD14^pos^ PMNs significantly reduced T cell proliferation (Fig. 3I, 3J). These results demonstrate that CD14^pos^ PMNs exhibit a MDSC-like phenotype and are functionally able to suppress T cell proliferation, potentially contributing to the establishment of an immunosuppressive microenvironment in the lungs of DEP-exposed mice.

### Chronic exposure to DEP induces an immunosuppressive microenvironment in the lung

**Fig. 4:**
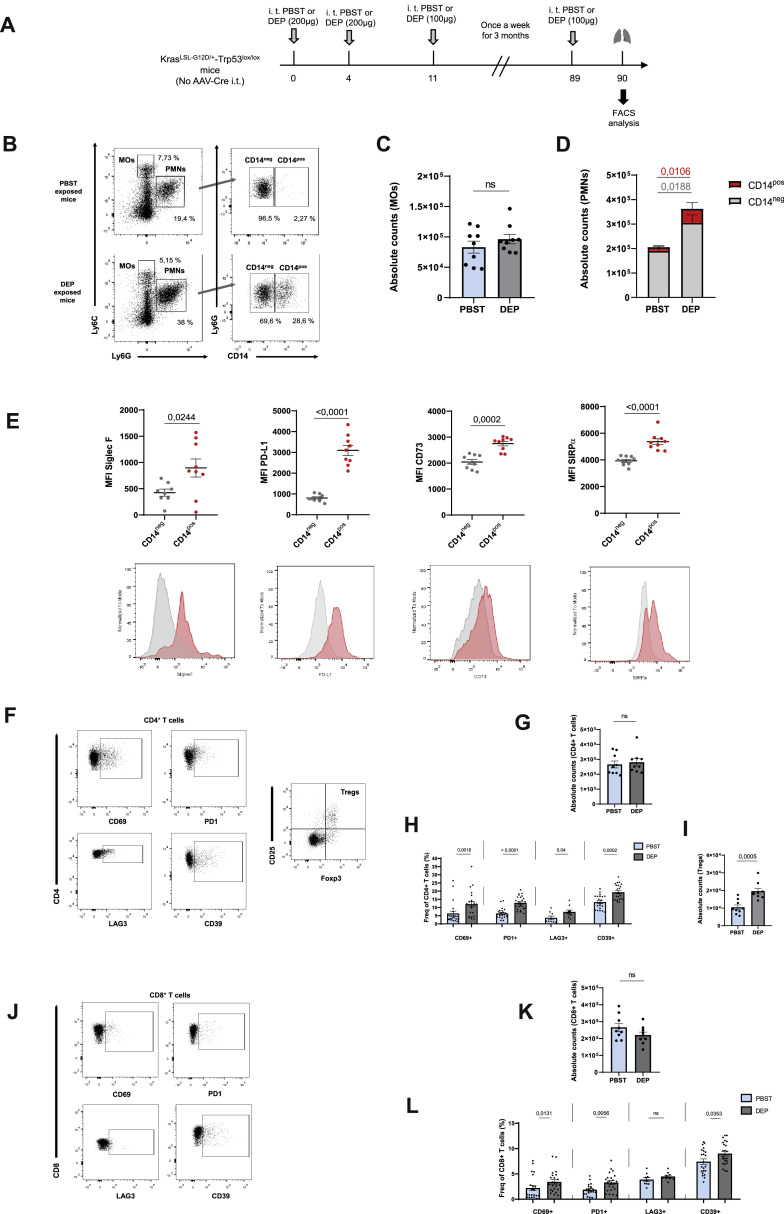
**Chronic exposure to DEP induces an immunosuppressive microenvironment in the lung** (A) Experimental exposure protocol. (B) Gating strategy used for the identification of MOs, PMNs and their subsets in lungs of PBST or DEP exposed mice. Absolute counts of (C) MOs (D) CD14^neg^ and CD14^pos^ PMN. (E) Mean fluorescence intensity (MFI) quantification of selected markers and representative flow cytometry plots of CD14^neg^ and CD14^pos^ PMNs (*n* = 8 to 9 mice per group, one experiment). Representative flow cytometry plots of (F) CD4+ *T* cells and (J) CD8+ *T* cells, absolute counts of (G) CD4+ *T* cells, (I) Tregs and (K) CD8+ *T* cells, frequency (%) of indicated markers present in (H) CD4+ *T* cells and (L) CD8+ *T* cells from PBST and DEP exposed mice. Bar graphs represent the mean ± SEM of up to three pooled experiments (*n* = 8-9 mice per group). Statistical significance was assessed using Mann-Whitney test.

We next investigated whether chronic DEP exposure, which may better reflect real-world environmental conditions, also promotes the emergence of CD14^pos^ PMNs with immunosuppressive properties in the lung (Fig. 4A). Consistent with our observations in the acute exposure model, DEP-exposed lungs showed a significant increase in numbers of PMNs compared to controls, along with a higher proportion of CD14^pos^ PMNs (Fig. 4B, 4D), while monocytes remained unchanged (Fig. 4B-C). Furthermore, CD14^pos^ PMNs exhibited significantly higher expression of Siglec-F and PD-L1, as well as regulatory markers, such as CD73, which is involved in adenosine production [10], and SIRPα, which is involved in the inhibition of phagocytosis of CD47-expressing target cells [11], compared to CD14^neg^ PMNs (Fig. 4E). In the spleen, on the other hand, no significant differences in PMN recruitment were observed and CD14^pos^ PMNs were not detected in either group (Fig. S5 A-B).

Although total CD4^+^ and CD8^+^ *T* cell numbers again remained unchanged in the lung following DEP chronic exposure (Fig. 4G, 4K), we observed alterations in the composition and activation state of T cell populations. Specifically, there was an increased proportion of Tregs (Fig. 4I), along with an upregulation of activation and exhaustion markers such as CD69, PD-1, LAG-3 (only in CD4^+^ T cells) and CD39. Similar to what we observed in the KP model, these alterations appear to be more pronounced in CD4^⁺^ T cells than in their CD8^⁺^ counterparts (Fig. 4F, 4H, 4J, 4L). These results demonstrate that chronic DEP exposure induces the emergence of MDSCs-like PMNs with a regulatory phenotype that may promote immunosuppression in the context of cancer, given the pivotal roles of PD-L1, CD73 and SIRPα in suppressing anti-tumour immune responses [[10], [11], [12]]. Furthermore, we observed that this regulatory and immunosuppressive signature extended to T cell populations, which showed increased expression of regulatory and activation/exhausted markers, changes that may impair effective T cell responses.

## Discussion

In this study, we investigated the mechanisms by which exposure to DEP may promote lung cancer progression. DEP are commonly found in cities, in places where diesel engines are used indoors or along roads. Previous studies have suggested that engine exhaust contributes to the development of lung tumours by inducing oxidative stress, inflammation and genotoxic effects [13,14]. However, the impact of DEP exposure on anti-tumour immune mechanisms remains elusive to date, and a comprehensive understanding of its effects on anti-tumour immunity is lacking.

Consistent with previous reports, our study supports the notion that exposure to DEP induces neutrophilic inflammation in the lungs, a phenomenon that has been directly linked to the promotion of lung metastasis [15]. Interestingly, similar mechanisms have been described in models of ozone exposure, where neutrophilic infiltration was also associated with enhanced metastatic dissemination [9]. Our results provide critical insights into the immunosuppressive signature of PMNs, particularly CD14^pos^ PMNs, following acute and chronic DEP exposure. CD14^pos^ PMNs in the lungs formed NETs as confirmed by the co-localization of citrullinated histone (Cit-H3) and extracellular DNA. NETs can contribute to tumour progression in multiple ways. Firstly, NETs are known to entrap circulating tumour cells and facilitate lung tissue invasion [9,16]. Furthermore, NETs can also suppress anti-tumour immune responses by coating tumour cells, thereby protecting them against cytotoxic CD8^+^ *T* cells and NK lymphocytes [17]. In addition, NETs can directly interact with infiltrating T cells by the expression of PD-L1 which engages PD-1 receptors on T cells. Such interaction promotes the upregulation of multiple inhibitory receptors, leading to diminished mitochondrial function and a shift toward a metabolically exhausted state [18].

Besides undergoing NETosis, DEP-recruited CD14^pos^ PMNs expressed key MDSC-associated genes, including Arg1, Nos2, Ptgs2, and Cd274 [19]. In addition, compared to their CD14^neg^ counterparts, CD14^pos^ PMNs exhibited increased expression of immunoregulatory and immunosuppressive cytokine such as IL-10 and TGFβ, along with Siglec-F expression, a marker associated with neutrophils displaying MDSC-like features [20]. CD14^pos^ PMNs demonstrated a functional ability to suppress T cell proliferation, which aligns with prior studies showing that these cells can promote immune suppression in cancer models, facilitating immune evasion and reducing anti-tumour responses [21]. The presence of CD14^pos^ PMNs in both cancer and non-cancer inflammatory conditions suggests that CD14 expression on tumour-infiltrating neutrophils reflects a broader inflammation-associated phenotype. These findings indicate that DEP-recruited CD14^pos^ PMNs acquire an immunosuppressive phenotype, combining both molecular and functional characteristics of PMN-MDSCs, which likely contribute to the establishment of a tumour-permissive immune microenvironment.

T cell exhaustion is a state of T cell dysfunction commonly observed in chronic infections and cancer. Exhausted T cells exhibit overexpression of inhibitory receptors and decreased effector cytokine production, resulting in a failure to eliminate cancer cells [22]. In the current study, chronic DEP led to the upregulation of inhibitory receptors commonly associated with an exhausted T cell phenotype in chronic settings, such as PD-1, LAG-3 and TIM-3, suggesting that chronic exposure to pollutants may drive T cell dysfunction [23,24]. Furthermore, other markers involved in immunosuppression, such as CD39, was also upregulated on T cells, along with CD73 on CD14^pos^ PMNs. CD39 and CD73 function together to convert extracellular ATP to adenosine, a molecule that inhibits T cell function and promotes Treg activity [25]. In line with this, the numbers of Tregs increased in the lungs of DEP-exposed mice.

Our findings, together with those of Hill et al., provide compelling evidence that airborne pollutants play a crucial role in lung cancer progression by shaping the tumour microenvironment rather than directly inducing mutations. Hill et al. demonstrated that PM_2.5_ exposure promotes EGFR- and KRAS-driven lung adenocarcinoma through an inflammatory response characterized by sustained influx of interstitial macrophages which upregulate PD-L1 and produce IL-1β. This pro-inflammatory environment facilitates the expansion of pre-existing oncogenic clones and reprograms EGFR -mutant alveolar type II (AT2) epithelial cells into a progenitor cell state, fostering tumour initiation and progression [26]. Our study extends these findings by showing that chronic DEP exposure promotes an immunosuppressive tumour milieu in KP mice, characterized by the appearance of immunosuppressive PMNs and the emergence of a dysfunctional T cell state.

Taken together, these findings underscore the multifaceted role of air pollution in lung cancer progression, involving both inflammatory and immunosuppressive pathways. Overall, our study highlights the impact of DEP on lung inflammation and its role in establishing an immunosuppressive, tumour-promoting lung microenvironment. Patients chronically exposed to high levels of PM may develop a tumour microenvironment enriched in immunosuppressive marks. As such, they could particularly benefit from immunotherapy targeting the CD47-SIRPα axis and CD73 molecules expressed on immunosuppressive neutrophils. In addition, the use of well-established immune checkpoint inhibitors, such as PD-1/PD-L1 blockers or LAG-3 antagonists, may hold enhanced therapeutic relevance in lung cancer patients exposed to high levels of air pollution. These approaches offer promise to counteract the immunosuppressive effects of DEP exposure and to restore effective anti-tumour immune responses.

## Funding sources

This work was funded by the FRS-FNRS (grant T.0036.25 and Télévie 7.6504.24) (Belgium), the Foundation against cancer (PDR grant 2024.187) (Belgium), the Fondation Léon Fredericq (University of Liege, Belgium), the Fonds spéciaux of the University of Liège (Belgium), the European Regional Development Fund (FEDER) - SYST-IMM project.

## CRediT authorship contribution statement

**Marie-Laure Delhez:** Conceptualization, Data curation, Formal analysis, Methodology, Validation, Writing – original draft, Writing – review & editing. **Maëlle Bosmans:** Formal analysis, Investigation, Methodology, Validation, Writing – review & editing. **Lucia Rodriguez Rodriguez:** Methodology, Validation, Writing – review & editing. **Alison Gillard:** Conceptualization, Investigation, Methodology. **Silvia Blacher:** Data curation, Formal analysis, Methodology. **Arnaud Blomme:** Investigation, Methodology, Writing – review & editing. **Pierre Close:** Methodology, Writing – review & editing. **Bénédicte Machiels:** Formal analysis, Methodology, Writing – review & editing. **Marie-Julie Nokin:** Formal analysis, Investigation, Methodology, Supervision, Validation, Writing – review & editing. **Didier Cataldo:** Conceptualization, Data curation, Formal analysis, Funding acquisition, Investigation, Methodology, Project administration, Resources, Supervision, Validation, Writing – original draft, Writing – review & editing.

## Declaration of competing interest

The authors declare that they have no known competing financial interests or personal relationships that could have appeared to influence the work reported in this paper.

## Appendix Supplementary materials

Image, application 1

Image, application 2

## References

[1] World Health Organization (2021). WHO global air quality guidelines: particulate matter (PM2.5 and PM10), ozone, nitrogen dioxide, sulfur dioxide and carbon monoxide. World Health Organization.

[2] Di Q., Wang Y., Zanobetti A., Wang Y., Koutrakis P., Choirat C., Dominici F., Schwartz J.D. (2017). Air pollution and mortality in the Medicare population. N. Engl. J. Med..

[3] IARC (2014). Diesel and gasoline engine exhausts and some Nitroarenes. IARC. Monogr. Eval. Carcinog. Risks. Hum..

[4] Zhang F., Xia Y., Su J., Quan F., Zhou H., Li Q., Feng Q., Lin C., Wang D., Jiang Z. (2024). Neutrophil diversity and function in health and disease. Signal. Transduct. Target. Ther..

[5] Wooding D.J., Ryu M.H., Li H., Alexis N.E., Pena O., Carlsten C., Canadian Respiratory Research Network (2020). Acute air pollution exposure alters neutrophils in never-smokers and at-risk humans. Eur. Respir. J..

[6] Hedrick C.C., Malanchi I. (2022). Neutrophils in cancer: heterogeneous and multifaceted. Nat. Rev. Immunol..

[7] Zhou J., Nefedova Y., Lei A., Gabrilovich D. (2018). Neutrophils and PMN-MDSC: their biological role and interaction with stromal cells. Semin. Immunol..

[8] Otsu N. (1979). A threshold selection method from gray-level histograms. IEEE Trans. Syst. Man Cybern.

[9] Rocks N., Vanwinge C., Radermecker C., Blacher S., Gilles C., Marée R., Gillard A., Evrard B., Pequeux C., Marichal T., Noel A., Cataldo D. (2019). Ozone-primed neutrophils promote early steps of tumour cell metastasis to lungs by enhancing their NET production. Thorax..

[10] Glodde N., Bald T., van den Boorn-Konijnenberg D., Nakamura K., O’Donnell J.S., Szczepanski S., Brandes M., Eickhoff S., Das I., Shridhar N., Hinze D., Rogava M., van der Sluis T.C., Ruotsalainen J.J., Gaffal E., Landsberg J., Ludwig K.U., Wilhelm C., Riek-Burchardt M., Müller A.J., Gebhardt C., Scolyer R.A., Long G.V., Janzen V., Teng M.W.L., Kastenmüller W., Mazzone M., Smyth M.J., Tüting T., Hölzel M. (2017). Reactive neutrophil responses dependent on the receptor tyrosine kinase c-MET limit cancer immunotherapy. Immunity.

[11] Zheng W., Zhu Y., Chen X., Zhao J. (2021). CD73 expression in myeloid-derived suppressor cells is correlated with clinical stages in head and neck squamous cell carcinomas. Ann. Transl. Med..

[12] Huang C., Wang X., Wang Y., Feng Y., Wang X., Chen S., Yan P., Liao J., Zhang Q., Mao C., Li Y., Wang L., Wang X., Yi W., Cai W., Chen S., Hong N., He W., Chen J., Jin W. (2024). Sirpα on tumor-associated myeloid cells restrains antitumor immunity in colorectal cancer independent of its interaction with CD47. Nat. Cancer.

[13] Lee J.W., Lee H.J., Lee Y.-J., Lim Y., Sim W.J., Jang J.-H., Heo H.-R., Lim H.J., Jung J.-W., Kim J.S. (2021). Determination of genotoxicity attributed to diesel exhaust particles in normal Human embryonic lung cell (WI-38) line. Biomolecules.

[14] Sakurai Y., Oba E., Honda A., Tanaka H., Takano H., Akita H. (2024). The stress-responsive cytotoxic effect of diesel exhaust particles on lymphatic endothelial cells. Sci. Rep..

[15] Li W., Liu T., Xiong Y., Lv J., Cui X., He R. (2018). Diesel exhaust particle promotes tumor lung metastasis via the induction of BLT1-mediated neutrophilic lung inflammation. Cytokine.

[16] Cools-Lartigue J., Spicer J., Najmeh S., Ferri L. (2014). Neutrophil extracellular traps in cancer progression. Cell Mol. Life Sci..

[17] Teijeira Á., Garasa S., Gato M., Alfaro C., Migueliz I., Cirella A., de Andrea C., Ochoa M.C., Otano I., Etxeberria I., Andueza M.P., Nieto C.P., Resano L., Azpilikueta A., Allegretti M., de Pizzol M., Ponz-Sarvisé M., Rouzaut A., Sanmamed M.F., Schalper K., Carleton M., Mellado M., Rodriguez-Ruiz M.E., Berraondo P., Perez-Gracia J.L., Melero I. (2020). CXCR1 and CXCR2 chemokine receptor agonists produced by tumors induce neutrophil extracellular traps that interfere with immune cytotoxicity. Immunity.

[18] Kaltenmeier C., Yazdani H.O., Morder K., Geller D.A., Simmons R.L., Tohme S. (2021). Neutrophil extracellular traps promote T cell exhaustion in the tumor microenvironment. Front. Immunol..

[19] Li K., Shi H., Zhang B., Ou X., Ma Q., Chen Y., Shu P., Li D., Wang Y. (2021). Myeloid-derived suppressor cells as immunosuppressive regulators and therapeutic targets in cancer. Signal. Transduct. Target. Ther..

[20] Engblom C., Pfirschke C., Zilionis R., Da Silva Martins J., Bos S.A., Courties G., Rickelt S., Severe N., Baryawno N., Faget J., Savova V., Zemmour D., Kline J., Siwicki M., Garris C., Pucci F., Liao H.-W., Lin Y.-J., Newton A., Yaghi O.K., Iwamoto Y., Tricot B., Wojtkiewicz G.R., Nahrendorf M., Cortez-Retamozo V., Meylan E., Hynes R.O., Demay M., Klein A., Bredella M.A., Scadden D.T., Weissleder R., Pittet M.J. (2017). Osteoblasts remotely supply lung tumors with cancer-promoting SiglecF ^high^ neutrophils. Science.

[21] Veglia F., Hashimoto A., Dweep H., Sanseviero E., De Leo A., Tcyganov E., Kossenkov A., Mulligan C., Nam B., Masters G., Patel J., Bhargava V., Wilkinson P., Smirnov D., Sepulveda M.A., Singhal S., Eruslanov E.B., Cristescu R., Loboda A., Nefedova Y., Gabrilovich D.I. (2021). Analysis of classical neutrophils and polymorphonuclear myeloid-derived suppressor cells in cancer patients and tumor-bearing mice. J. Exp. Med..

[22] Pauken K.E., Wherry E.J. (2015). Overcoming T cell exhaustion in infection and cancer. Trends Immunol..

[23] Roussel M., Le K.-S., Granier C., Llamas Gutierrez F., Foucher E., Le Gallou S., Pangault C., Xerri L., Launay V., Lamy T., Tartour E., Olive D., Fest T. (2021). Functional characterization of PD1+TIM3+ tumor-infiltrating T cells in DLBCL and effects of PD1 or TIM3 blockade. Blood Adv..

[24] Ciraolo E., Althoff S., Ruß J., Rosnev S., Butze M., Pühl M., Frentsch M., Bullinger L., Na I.-K. (2022). Simultaneous genetic ablation of PD-1, LAG-3, and TIM-3 in CD8 T cells delays tumor growth and improves survival outcome. Int. J. Mol. Sci..

[25] Xia C., Yin S., To K.K.W., Fu L. (2023). CD39/CD73/A2AR pathway and cancer immunotherapy. Mol. Cancer.

[26] Hill W., Lim E.L., Weeden C.E., Lee C., Augustine M., Chen K., Kuan F.-C., Marongiu F., Evans E.J., Moore D.A., Rodrigues F.S., Pich O., Bakker B., Cha H., Myers R., van Maldegem F., Boumelha J., Veeriah S., Rowan A., Naceur-Lombardelli C., Karasaki T., Sivakumar M., De S., Caswell D.R., Nagano A., Black J.R.M., Martínez-Ruiz C., Ryu M.H., Huff R.D., Li S., Favé M.-J., Magness A., Suárez-Bonnet A., Priestnall S.L., Lüchtenborg M., Lavelle K., Pethick J., Hardy S., McRonald F.E., Lin M.-H., Troccoli C.I., Ghosh M., Miller Y.E., Merrick D.T., Keith R.L., Al Bakir M., Bailey C., Hill M.S., Saal L.H., Chen Y., George A.M., Abbosh C., Kanu N., Lee S.-H., McGranahan N., Berg C.D., Sasieni P., Houlston R., Turnbull C., Lam S., Awadalla P., Grönroos E., Downward J., Jacks T., Carlsten C., Malanchi I., Hackshaw A., Litchfield K., DeGregori J., Jamal-Hanjani M., Swanton C. (2023). Lung adenocarcinoma promotion by air pollutants. Nature.

