# Supplementary material for: Exposure to diesel particulates induces an immunosuppressive microenvironment that promotes the progression of lung cancer
Source: Neoplasia. 2025 Nov 21;71:101255. doi: 10.1016/j.neo.2025.101255 (PMC12681886; doi:10.1016/j.neo.2025.101255)
Supplement: Supplementary file 1 [file mmc1.docx]

| REAGENT or RESOURCE SOURCE IDENTIFIER | | |
| --- | --- | --- |
| Antibodies | | |
| PerCP/Cyanine5.5 anti-mouse Ly-6G Antibody (Clone 1A8) | Biolegend | 127615; RRID : AB_1877272 |
| PE anti-mouse CD64 (FcγRI) Antibody (Clone X54-5/7.1) | Biolegend | 139303; RRID : AB_10613467 |
| PE/Dazzle™ 594 anti-mouse CD8a Antibody (Clone 53-6.7) | Biolegend | 100761; RRID : AB_2564026 |
| PE-Cy™7 anti-Mouse CD11c (Clone HL3) | BD Biosciences | 558079; RRID : AB_647251 |
| APC anti-mouse CD170 (Siglec-F) Antibody (Clone S17007L) | Biolegend | 155507; RRID : AB_2750236 |
| Brilliant Violet 650™ anti-mouse Ly-6C Antibody (Clone HK1.4) | Biolegend | 128049; RRID : AB_2800630 |
| Brilliant Violet 785™ anti-mouse/human CD11b Antibody (Clone M1/70) | Biolegend | 101243; RRID : AB_2561373 |
| BUV395 anti-Mouse CD45 (Clone 30-F11) | BD Biosciences | 564279; RRID : AB_2651134 |
| Spark UV™ 387 anti-mouse CD45 Antibody (Clone 30-F11) | Biolegend | 103187; RRID : AB­_3083254 |
| BUV737 Rat Anti-Mouse CD4 (Clone RM4-5) | BD Biosciences | 612843; RRID : AB_2870165 |
| PE anti-mouse/human CD11b Antibody (Clone M1/70) | Biolegend | 101207; RRID : AB_312790 |
| PE/Cyanine7 anti-mouse CD45 Antibody (Clone 30-F11) | Biolegend | 103113; RRID : AB_312978 |
| APC anti-mouse CD14 Antibody (Clone Sa14-2) | Biolegend | 123311; RRID : AB_940574 |
| Brilliant Violet 421™ anti-mouse Ly-6G Antibody (Clone 1A8) | Biolegend | 127627; RRID : AB_10897944 |
| Brilliant Violet 785™ anti-mouse Ly-6C Antibody (Clone HK1.4) | Biolegend | 128041; RRID : AB_2565852 |
| PE-CF594 anti-mouse Siglec-F (Clone E50-2440) | BD Biosciences | 562757; RRID : AB_2687994 |
| Brilliant Violet 711™ anti-mouse CD64 (FcγRI) Antibody (Clone X54-5/7.1) | Biolegend | 139311; RRID : AB_2563846 |
| PE anti-mouse CD3ε Antibody (Clone KT3.1.1) | Biolegend | 155607; RRID : AB_2750433 |
| FITC anti-mouse CD3ε Antibody (Clone 145-2C11) | Biolegend | 100306; RRID : AB­_312670 |
| APC anti-mouse CD4 Antibody (Clone RM4-5) | Biolegend | 100515; RRID : AB_312718 |
| FITC anti-mouse CD8a Antibody (Clone 53-6.7) | Biolegend | 100705; RRID : AB_312744 |
| CD3 monoclonal antibody (Clone 17A2) | eBioscience | 16-0032-82; RRID : AB_468851 |
| PerCP/Cyanine5.5 anti-mouse/human CD11b Antibody (Clone M1/70) | Biolegend | 101227; RRID : AB_893233 |
| APC/Fire™ 750 anti-mouse Ly-6G Antibody (Clone 1A8) | Biolegend | 127651; RRID : AB_2616732 |
| Brilliant Violet 711™ anti-mouse CD274 (B7-H1, PD-L1) Antibody (Clone 10F.9G2) | Biolegend | 124319; RRID : AB_2563619 |
| PE/Dazzle™ 594 anti-mouse CD172a (SIRPα) Antibody (Clone P84) | Biolegend | 144015; RRID : AB_2565279 |
| PE anti-mouse CD73 Antibody (Clone TY/11.8) | Biolegend | 127205; RRID : AB_1089065 |
| Brilliant Violet 421™ anti-mouse CD170 (Siglec-F) Antibody (Clone S17007L) | Biolegend | 155509; RRID : AB_2810421 |
| Alexa Fluor® 700 anti-mouse CD45 Antibody (Clone 30-F11) | Biolegend | 103127; RRID : AB_4937140 |
| PE anti-mouse CD3 Antibody (Clone 17 A2) | Biolegend | 100205; RRID : AB_312662 |
| APC/Fire™ 750 anti-mouse CD4 Antibody (Clone RM4-5) | Biolegend | 100567; RRID : AB_2629698 |
| PerCP/Cyanine5.5 anti-mouse CD8a Antibody (Clone 53-6.7) | Biolegend | 100733; RRID : AB_2075239 |
| Brilliant Violet 785™ anti-mouse CD69 Antibody (Clone H1.2F3) | Biolegend | 104543; RRID : AB_2629640 |
| PE-Cyanine7 anti-mouse CD279 (PD-1) monoclonal Antibody (Clone J43) | eBioscience | 25-9985-80; RRID : AB_10853805 |
| APC anti-mouse CD39 Antibody (Clone Duha59) | Biolegend | 143809; RRID : AB_2750319 |
| Brilliant Violet 711™ anti-mouse CD25 Antibody (Clone PC61) | Biolegend | 102049; RRID : AB_2564130 |
| eFluor™ 450 anti-mouse FOXP3 monoclonal Antibody (Clone FJK-16s) | eBioscience | 48-5773-82; RRID : AB_1518812 |
| PE-Cyanine7 anti-mouse Arginase 1 monoclonal Antibody (Clone A1exF5) | eBioscience | 25-3697-82 ; RRID : AB_2734841 |
| FITC Mouse anti-Ki67 Set | BD Biosciences | 556026; RRID : AB_396302 |
| APC anti-mouse NK-1.1 Antibody (Clone S17016D) | Biolegend | 156505; RRID : AB_2876525 |
| eFluor™ 450 anti-mouse CD4 monoclonal Antibody (Clone RM4-5) | eBioscience | 48-0042-82; RRID : AB_1272194 |
| Brilliant Violet 711™ anti-mouse CD223 (LAG-3) Antibody (Clone C9B7W) | Biolegend | 125243; RRID : AB_2876450 |
| Purified anti-mouse CD16/32 Antibody (Clone 93) | Biolegend | 101301; RRID : AB_312800 |
| Anti-Histone H3 (citrulline R2 + R8 + R17) antibody | Abcam | Ab5103 |
| Anti-Human/Mouse Myeloperoxidase/MPO antibody | R&D Systems | AF3667 |
| Anti-Ki67 antibody | Abcam | Ab16667 |
| Donkey anti-Rabbit IgG (H+L) Highly Cross-Adsorbed Secondary Antibody, Alexa Fluor™ 555 | ThermoFisher Scientific | A-31572 |
| Donkey anti-Goat IgG (H+L) Cross-Adsorbed Secondary Antibody, Alexa Fluor™ 488 | ThermoFisher Scientific | A-11055 |
| Anti-Ly-6G MicroBeads UltraPure, mouse | Miltenyi Biotec | 130-120-337 |
| Chemicals | | |
| Zombie Aqua™ Fixable Viability Kit | Biolegend | 423101 |
| Zombie NIR™ Fixable Viability Kit | Biolegend | 423105 |
| Zombie Violet™ Fixable Viability Kit | Biolegend | 423113 |
| CellTrace™ Violet Cell Proliferation Kit | ThermoFisher Scientific | C34557 |
| SYTOX™ Blue Dead Cell Stain | ThermoFisher Scientific | S34857 |
| Hoechst 33342, trihydrochloride, trihydrate | ThermoFisher Scientific | H3570 |
| Pan T cells Isolation Kit II, mouse | Miltenyi Biotec | 130-095-130 |
| Cyto-Fast Fix/Perm Buffer Set | Biolegend | 426803 |
| Foxp3/Transcription Factor Staining Buffer Set | eBioscience | 00-5523-00 |
