# Supplementary material for: Exposure to diesel particulates induces an immunosuppressive microenvironment that promotes the progression of lung cancer
Source: Neoplasia. 2025 Nov 21;71:101255. doi: 10.1016/j.neo.2025.101255 (PMC12681886; doi:10.1016/j.neo.2025.101255)
Supplement: Supplementary file 2 [file mmc2.pdf]

Supplementary material and methods

## Intratracheal instillation of DEP into mouse lungs

DEP were provided by a Standard Reference Material (SRM) produced and certified by the National Institute of Standards and Technology (NIST) (Sigma Aldrich; NIST1650b; 1333-86-4). DEP (4mg/ml) were resuspended in sterile PBS with 0,05% Tween-20 (Sigma-Aldrich) using sonication. Mice were briefly anesthetized using 2,5% isoflurane/O2 mixture followed by intratracheal administration of 100µg or 200µg DEP in a volume of 50µl Phosphate-Buffered Saline Tween-20 solution (PBST) at the indicated time points.

*Acute exposure model*

C57BL/6 mice were intratracheally (i.t.) administered 200 µg of DEP or PBST on days 0 and 4, followed by euthanasia on day 5.

*Chronic exposure model*

In this model, Cre recombination was never induced, and *Kras*$^{LSL\text{-}G12D/+}$-*Trp53*$^{lox/lox}$ mice (KP) did not develop spontaneous lung tumours. Non-recombined KP mice received two i.t. doses of 200 µg DEP or PBST on days 0 and 4, followed by weekly administration of 100 µg DEP or PBST. Mice were euthanized 90 days after the first i.t. administration.

*Transgenic mouse model of lung adenocarcinoma*

In this model, *Kras*$^{LSL\text{-}G12D/+}$-*Trp53*$^{lox/lox}$ mice initially followed the same protocol of DEP instillation as the chronic exposure model. In addition, Cre-mediated recombination was initiated at day 5 by intratracheal administration of an AAV-Cre vector (AAV6.2ff-Spb-Cre; 5,8 x $10^{12}$ virus particles per 100 µl) to induce spontaneous tumorigenesis. Mice were euthanized 90 days after the AAV-Cre administration, and tumour burden was assessed by histological analysis using hematoxylin and eosin (H&E) staining.

**Lung cell and spleen cell isolation**

Mice were perfused with ice-cold PBS via the right ventricle after sacrifice. The lungs were then excised, finely minced with scalpel blades, and subjected to enzymatic digestion in HBSS medium containing collagenase D (1 mg/ml, Roche) and deoxyribonuclease I (50 µg/ml, Roche) at 37°C for 1 hour with intermittent shaking. Red blood cells were removed resuspending cells in 1 ml of 1x RBC lysis buffer (Thermo Fisher Scientific) incubated for 5 minutes at room temperature, then washed with PBS containing 2% FBS and 2.5 mM EDTA. Lung cells were subsequently filtered through a 70 µM cell strainer (Corning), resulting in a single-cell suspensions that was kept at 4°C for all steps if not stated otherwise.

Spleens were crashed manually with the embole of a syringer. Red blood cells were removed resuspending cells 2x in 1 ml of 1x RBC lysis buffer (Thermo Fisher Scientific) incubated for 5 minutes at room temperature, then washed with PBS containing 2% FBS and 2.5 mM EDTA. Spleen cells were subsequently filtered through a 70 µM cell strainer (Corning), resulting in a single-cell suspensions that was kept at 4°C for all follow up steps if not stated otherwise.

**Quantitative real time polymerase chain reaction (qRT-PCR)**

Total RNA was extracted from sorted $CD14^{neg}$ and $CD14^{pos}$ PMNs using the ReliaPrep RNA Cell Miniprep System kit (Promega) according to the manufacturer's instructions. cDNA was produced using FastGene Scriptase II cDNA 5x ReadyMix (Nippon Genetics EUROPE) and the reverse transcription reaction was then subjected to PCR amplification using FastStart Universal SYBR Green Master (Roche). PCR signals were recorded on a QuantStudio 3 Real-Time PCR System (ThermoFisher) and analysed using the ThermoFisher Design and Analysis Software 2.6.0. Primer sets

included murine iNOS-Fwd (5'-GTTCTCAGCCCAACAATACAAGA-3'), murine iNOS-Rv (5'-GTGGACGGGTCGATGTCAC -3'), murine CD274-Fwd (5'-GCTCCAAAGGACTTGTACGTG-3'), murine CD274-Rv (5'-TGATCTGAAGGGCAGCATTTC-3'), murine PTGS2-Fwd (5'-TGAGCAACTATTCCAAACCAGC-3'), murine PTGS2-Rv (5'-GCACGTAGTCTTCGATCACTATC-3'), murine ARG1-Fwd (5'-CTCCAAGCCAAAGTCCTTAGAG-3'), murine ARG1-Rv (5'-AGGAGCTGTCATTAGGGACAT-3'), murine IL10-Fwd (5'-GCTCTTACTGACTGGCATGAG-3'), murine IL10-Rv (5'-CGCAGCTCTAGGAGCATGTG-3'), murine TGFβ-Fwd (5'-CGTCACTGGAGTTGTACGGCAG-3'), murine TGFβ-Rv (5'-CGTTTGGGGCTGATCCCGTTG-3').

**Identification of NETs In Vivo and In Vitro**

In our *in vivo* experiments, 4 μm sections of paraffin-embedded mouse lungs were prepared and mounted on glass slides. Following dewaxing, antigen retrieval was conducted using EDTA buffer (Dako), and the sections were permeabilized with 0.5% Triton X-100 for 2 minutes. The specimens were then blocked with Animal-Free Blocking Solution (Cell Signaling). The sections were incubated with primary antibodies: anti-citrullinated-histone H3 (1:100; Abcam ab5103) and anti-myeloperoxidase (MPO) (1:60; R&D Systems AF3667). Citrullinated histone H3 is a specific marker of NET formation, as histone citrullination by PAD4 is a key step in chromatin decondensation during NETosis. Detection was performed using Alexa Fluor 555 donkey anti-rabbit (1:200; ThermoFisher A-31572) and Alexa Fluor 488 donkey anti-goat (1:200; ThermoFisher A-11055) secondary antibodies for 1 hour at room temperature. Hoechst staining was used to detect DNA.

In our *in vitro* experiments, sorted CD14$^{neg}$ and CD14$^{pos}$ PMNs (2x10$^5$) were plated and allowed to adhere to Poly-D-lysine-coated slides (Sigma-Aldrich) in DMEM without FBS at 37°C in a 5% CO2 environment for 16 hours. The slides were fixed in 4% paraformaldehyde (PFA), then permeabilized, blocked, and incubated with the previously described primary and secondary antibodies at the same concentrations. Visualization was performed using an Olympus Slideview VS200. Fluorescence microscopy images were analysed with Image J software to count the number of NETs per one hundred neutrophils. To quantify NET-DNA in bronchoalveolar lavage fluid (BALF), a PicoGreen assay kit (Invitrogen) was employed.

**Immunohistochemistry**

Tumour-bearing lungs of KP mice were perfused post-mortem by PBS injection through the right ventricle of the heart. The tissues were then fixed overnight in 10% formalin and subsequently transferred to 70% ethanol for storage until further processing. For histological analysis, the tissues were paraffin-embedded, sectioned, and stained with hematoxylin and eosin (H&E).

An original image analysis algorithm was implemented to automatically perform image processing and measurements using the Image Processing Toolbox of MATLAB 9.13 (R2022b) software (MathWorks, Inc.). This method allowed the determination of tumour size distribution through the following steps:

1. Image acquisition: Histological section images were acquired in full-colour red, green, and blue (RGB) space. In these images, tumours appeared as dark red regions clearly contrasted against a light red background. Image processing was then carried out using only the red channel.

2. Image binarization: To determine tumour size, the red channel images were binarized using an automatic thresholding technique (8). In the resulting binary images,

all pixels corresponding to the objects of interest (i.e., dark red tumours) were assigned a value of 1, while background pixels were assigned a value of 0. Binary images also contained non-tumour structures, such as epithelial cells surrounding the alveoli and. these were removed using morphological filtering. The resulting binary images were then systematically compared with the original images and manually corrected when necessary.

3. Tumour labelling and measurement: Each tumour in the binary images was labelled, and the number of pixels in each tumour was recorded. These pixel counts were then multiplied by the calibrated size of a single pixel to calculate tumour areas.

4. Size distribution analysis: A histogram of tumour sizes was generated for each mouse, and the mean histogram was calculated for each experimental condition.

Ki67 staining was performed in paraffin-embedded tissue sections that were first deparaffinized and rehydrated. Antigen retrieval was conducted using Target Retrieval Solution (Citrate pH 6, Dako) under heat-induced conditions. To inhibit endogenous peroxidase activity, sections were incubated in 3% hydrogen peroxide ($H_2O_2$) in distilled water for 20 minutes at room temperature. Subsequently, sections were blocked with an Animal-Free Blocking Solution (Cell Signaling) for 1 hour at room temperature. The sections were then incubated with the primary antibody, Ki67 (1:100, Abcam ab1667), for 1 hour at room temperature. Detection was carried out using the EnVision System-HRP, followed by incubation with the DAB substrate. Sections were counterstained with Hematoxylin-eosin, and images were captured using a NanoZoomer 2.0-HT slide scanner system (Hamamatsu) with NanoZoomer Digital Pathology software. Cellular proliferation was assessed by quantifying the density of Ki67 staining in tumour nodules, defined as the area occupied by Ki67-positive regions divided by the total area of the whole image. Ki67 regions were binarized from the red

channel of the original RGB images, using the same method described above. From the resulting binary images, the total number of pixels corresponding to Ki67 staining was calculated and normalized to the total image area.

**Figure S1. Flow cytometry gating strategy used to identify immune cells**. Example of flow cytometry gating strategy used to identify alveolar macrophages (AMs), eosinophils, neutrophils, monocytes (MOs), interstitial macrophages (IMs), CD4+ T cells, CD8+ T cells and dendritic cells (cDCs). Size gates were applied at the beginning of the gating strategy to exclude debris and doublets and were followed by live cell discrimination.

**Figure S2. Flow cytometry gating strategy used to identify different subsets of PMNs**. Example of flow cytometry gating strategy used to identify $CD14^{neg}$ and $CD14^{pos}$ PMNs populations. Size gates were applied at the beginning of the gating strategy to exclude debris and doublets and were followed by live cell discrimination.

**Figure S3. Flow cytometry gating strategy used to identify activation/exhaustion and immunosuppressive markers on T cells.** Example of flow cytometry gating strategy used to identify T regs and T cells expressing PD1, CD69, CD39, TIM3 and LAG3. Size gates were applied at the beginning of the gating strategy to exclude debris and doublets and were followed by live cell discrimination.

**Figure S4**. **Acute DEP-exposure doesn't induce $CD14^{pos}$ PMNs in the spleen** (A) Gating strategy used for the identification of MOs, PMNs and their subsets in spleen of PBST or DEP- exposed mice according to the exposure protocol in Fig. 2A. Absolute

counts of (B) MOs, (C) CD14$^{neg}$ and CD14$^{pos}$ PMNs recruited in the spleen of PBST or DEP-exposed mice (n = 5 mice per group, one experiment). Data are presented as mean +/- SEM and Mann-Whitney test was used to compare the two groups.

**Figure S5**. **Chronic DEP-exposure doesn't induce CD14$^{pos}$ PMNs in the spleen** (A) Gating strategy used for the identification of MOs, PMNs and their subsets in spleen of PBST or DEP-exposed mice according to the exposure protocol in Fig. 4A. Absolute counts of (B) MOs and (C) CD14$^{neg}$ and CD14$^{pos}$ PMNs recruited in the spleen of PBST and DEP- exposed mice (n = 9 mice per group, one experiment). Data are presented as mean +/- SEM and Mann-Whitney test was used to compare the two groups.

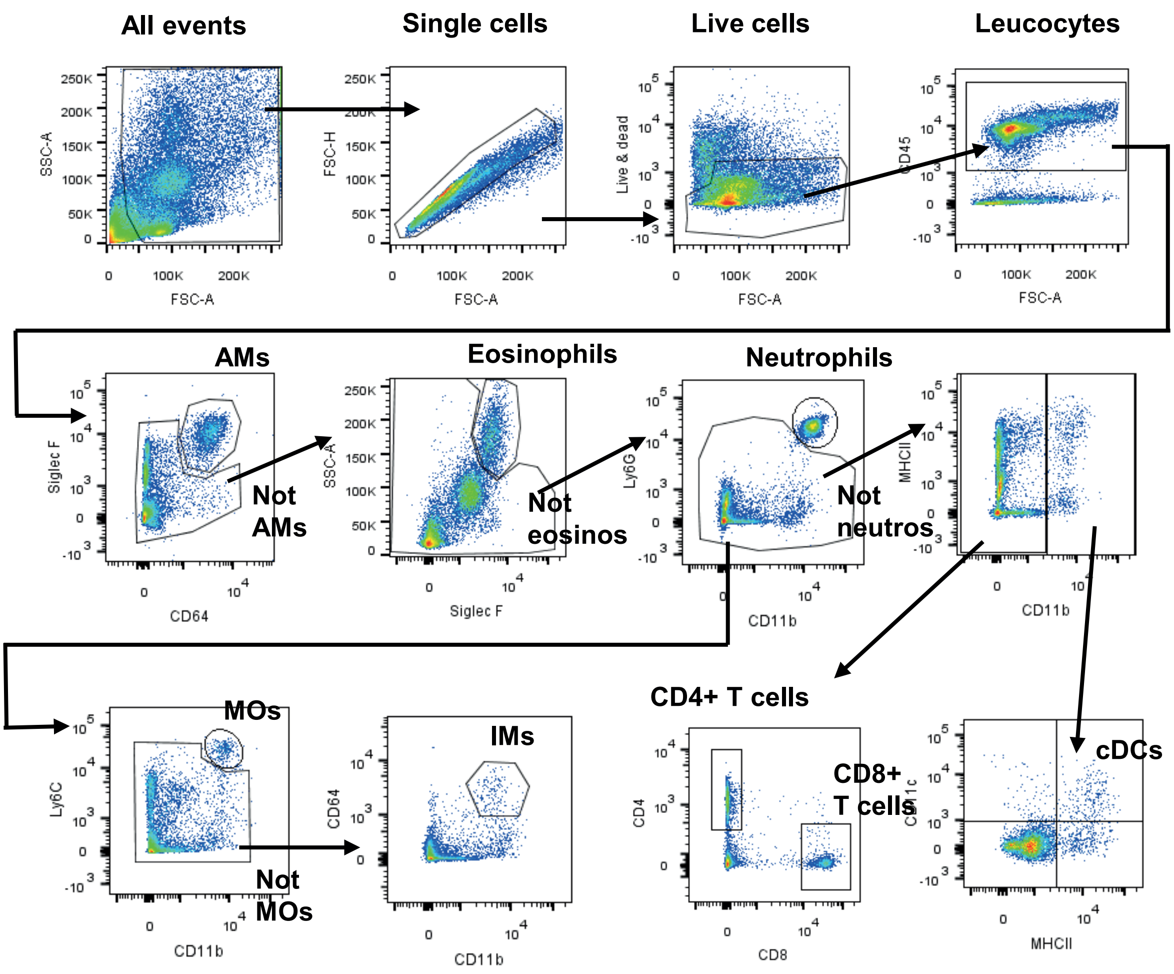
All events
Single cells
Live cells
Leucocytes
SSC-A
FSC-A
FSC-H
Live & dead
CD45
AMs
Not AMs
Eosinophils
Not eosinos
Neutrophils
Not neutros
Siglec F
CD64
SSC-A
Ly6G
CD11b
MHCII
MOs
Not MOs
IMs
CD4+ T cells
CD8+ T cells
cDCs
Ly6C
CD4
CD8
CD11c
MHCII

Figure S1

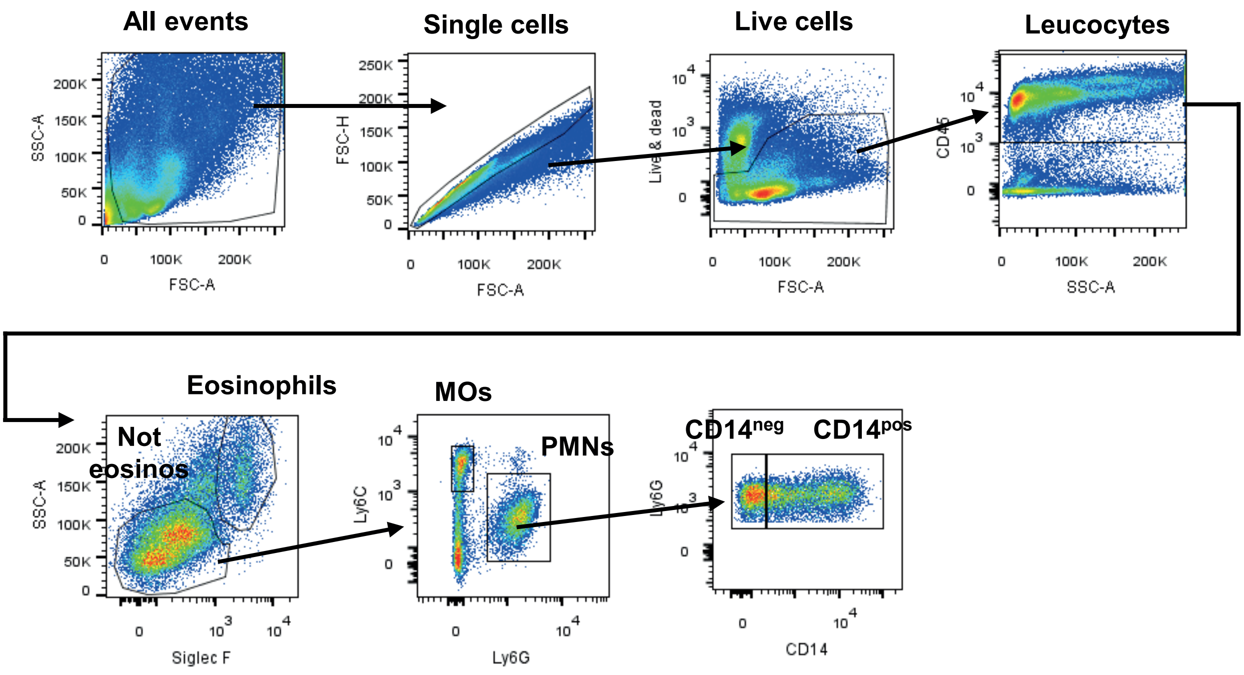
All events
Single cells
Live cells
Leucocytes
SSC-A
FSC-A
FSC-H
Live & dead
CD45
Eosinophils
Not eosinos
MOs
PMNs
CD14neg
CD14pos
Siglec F
Ly6C
Ly6G
CD14

Figure S2

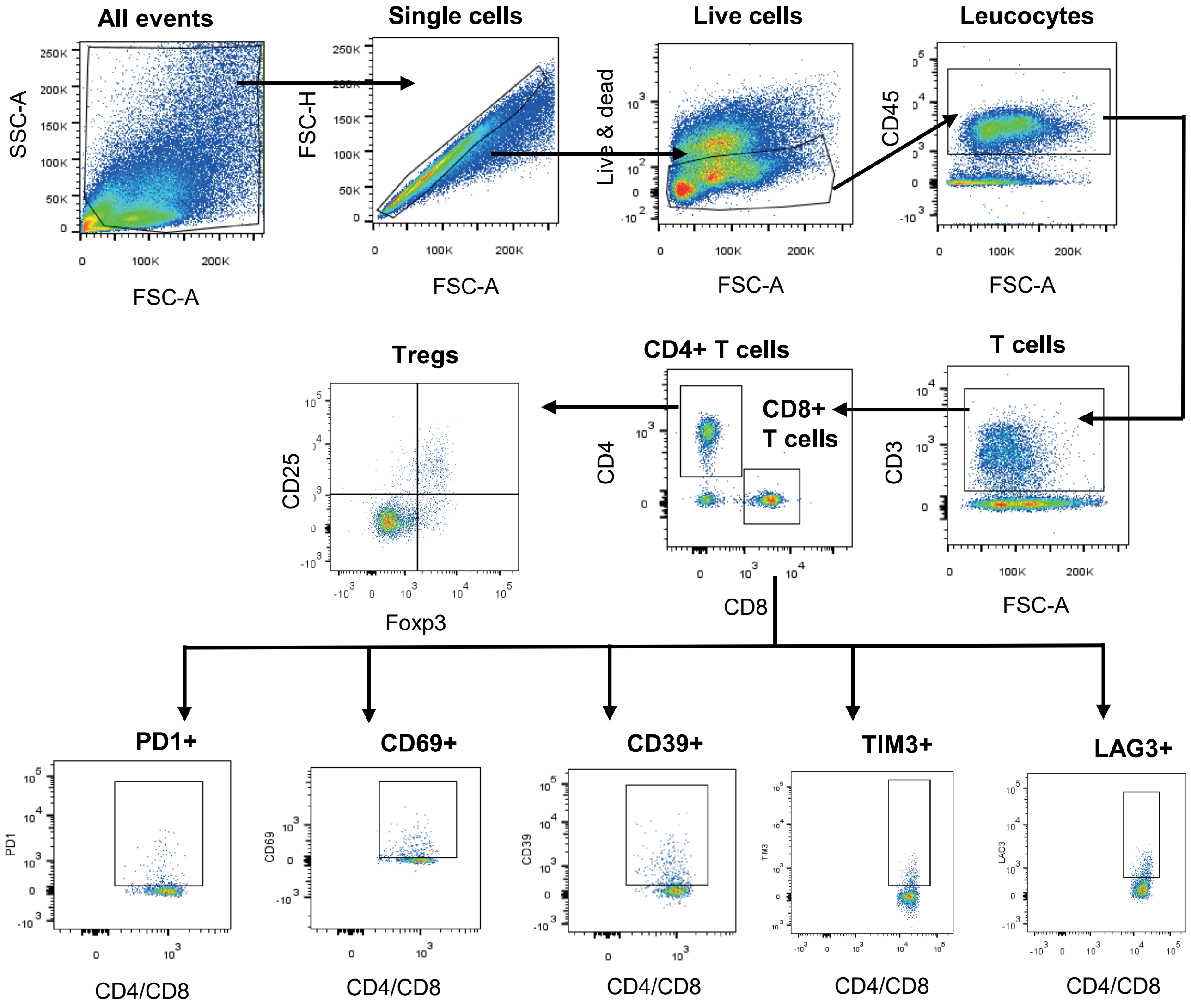
All events
SSC-A
FSC-A
Single cells
FSC-H
Live cells
Live & dead
Leucocytes
CD45
T cells
CD3
CD4+ T cells
CD8+
T cells
CD4
CD8
Tregs
CD25
Foxp3
PD1+
CD69+
CD39+
TIM3+
LAG3+
CD4/CD8

**Figure S3**

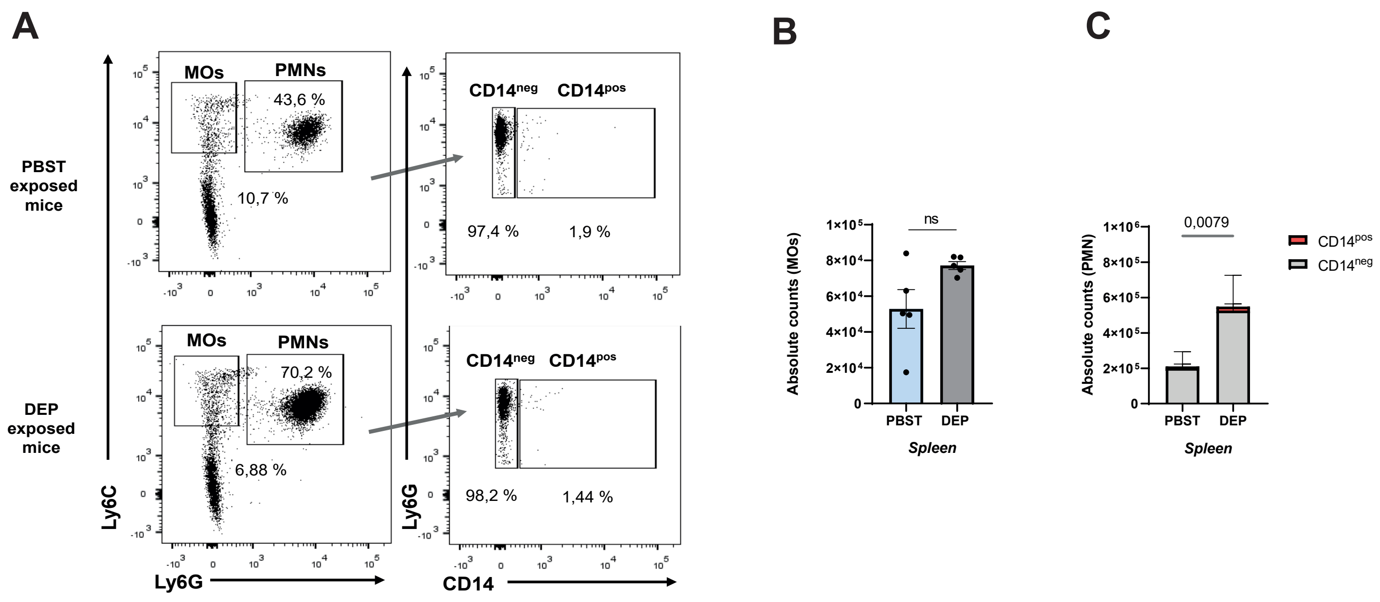
A
MOs
PMNs
43,6 %
10,7 %
PBST exposed mice
CD14neg
CD14pos
97,4 %
1,9 %
70,2 %
6,88 %
DEP exposed mice
98,2 %
1,44 %
Ly6C
Ly6G
CD14
B
Absolute counts (MOs)
ns
PBST
DEP
Spleen
C
Absolute counts (PMN)
0,0079
CD14pos
CD14neg
PBST
DEP
Spleen

**Figure S4**

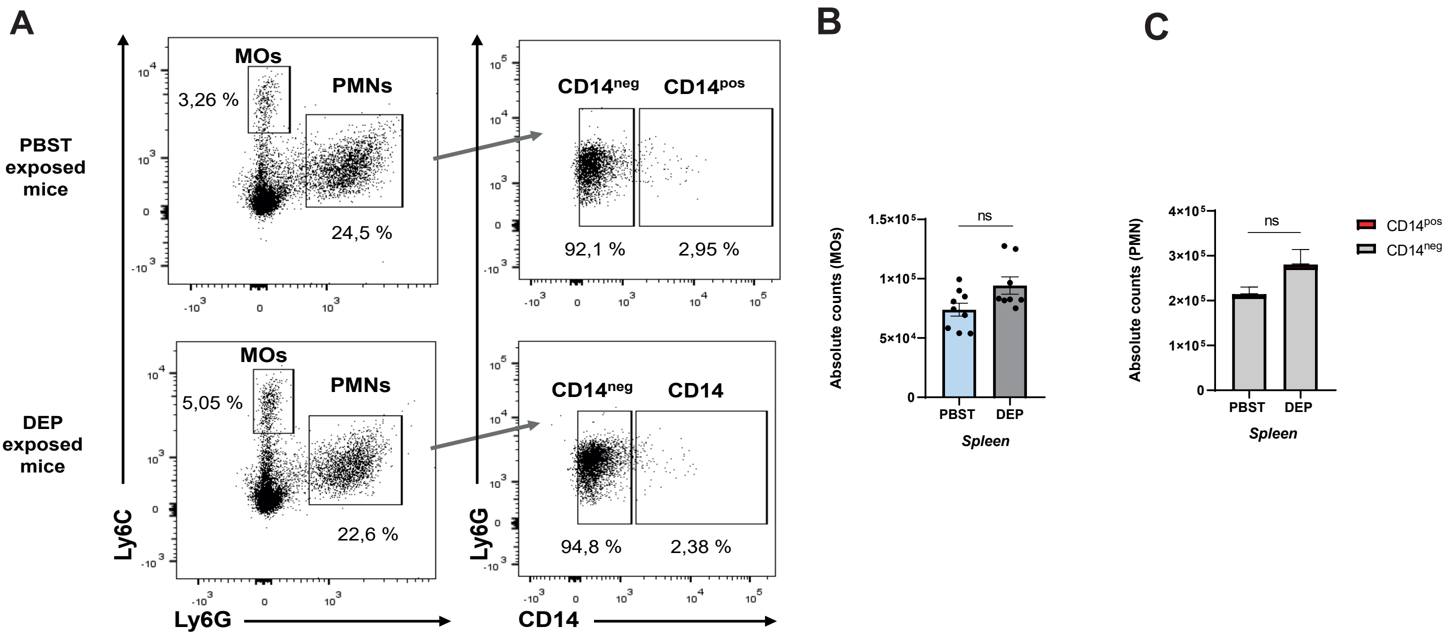
A
B
C
PBST exposed mice
DEP exposed mice
MOs
PMNs
3,26 %
24,5 %
5,05 %
22,6 %
CD14neg
CD14pos
CD14
92,1 %
2,95 %
94,8 %
2,38 %
Ly6C
Ly6G
CD14
Absolute counts (MOs)
Absolute counts (PMN)
ns
PBST
DEP
Spleen
CD14pos
CD14neg

# Figure S5
